# Collectanea Medica: Consisting of Anecdotes, Facts, Extracts, Illustrations, &c.

**Published:** 1823-12

**Authors:** 


### COLLECTANEA MEDICA:

CONSISTING OF

### ANECDOTES, FACTS, EXTRACTS, ILLUSTRATIONS, &c.

*Relating to the History or the Art of Medicine, and the Collateral Sciences.*

Floriferis, ut apes, in saltibus omnia libant,

Omnia nos, itidem, depascimur aurea dicta.

### Art. I.—*Extracts from "Medical Jurisprudence."* By J. A. Paris, M.D. &c. and J. S. M. Fonblanque, Esq.

*Of Impositions.—Feigned, or Simulated Diseases,* (concluded from page 394.)

*Vomiting of Blood.*—Sauvages relates the case of a young woman, who, to avoid the confinement of a convent, swallowed a quantity of bullock's blood, and vomited it up in the presence of a physician sent to examine her. Where such a trick is suspected, we have only to secure the patient from the necessary supplies, and the fraud is at once detected.

*Vomiting of Urine.*—Where this is asserted, we may safely pronounce the patient an impostor, for the event is physiologically impossible.

*Bloody Urine.*—An appearance of this nature is often produced in India, by eating the Indian fig *(cactus opuntia)*, or the fruit of the prickly pear, which imparts to the urine a blood-red colour. It has been also simulated by clandestinely pouring real blood, or colouring matter, into the night utensils. There is an old story of a boy who imposed on many by pretending to pass black urine; but, being confined, he was detected in an attempt to secrete an ink-bottle, which pointed out the mode of his imposture.

*Incontinence of Urine.*—The simulation of this affection may be detected by giving the patient a full dose of opium at night, without his knowledge, and introducing the catheter during sleep; or by taking him by surprise during the day, and introducing the same instrument; when, if he be an impostor, it will be found that the urine has not drained off *guttatim,* as it was secreted, but that the bladder possesses the power of retention. If the bed-clothes are not found wet after a full dose of opium, during the operation of which the patient has been suddenly awoke, we may also be satisfied that there is no incontinence. Foderé says that, if the penis is secured by a ligature, it will swell considerably in the real incontinence, in consequence of the urine running into the urethra; but that no such effect will happen if the disease be feigned.

*Gravel and Stone.*—All impositions upon this subject may be detected by chemical analysis. In general, it will be sufficient to saw the pretended calculus into two parts, when the absence of the characteristic structure will establish the fraud: it will frequently be found that they are small pebbles, or coarse siliceous sand. Mr. Wilson has related two instances of this kind, in which an attempt was made to practise on his credulity: "Many years ago, (says he,) when I resided in the house of Mr. Cruikshank, a person brought his son to that gentleman for surgical advice, asserting that the boy had long been cruelly afflicted with stone; in proof of which he produced several pieces of hard slaty substances, which he stated he had assisted the child in removing from the urethra. Upon my expressing an opinion that these were not urinary concretions, he pretended to be angry, and indignantly left the house, declaring that he would seek for a surgeon to perform the operation for the removal of the stone, whose humanity would not let him doubt the assertion of a father, who, though in poverty, would gladly sacrifice his own existence to save that of his son. A few days after this he brought back the boy, with a large piece of slate sticking in the urethra, which had torn the inner membrane, and, from the swelling it had produced, was with much difficulty removed. Wishing to detect the imposture, I persuaded him to leave the boy in Mr. Cruikshank's house, under the pretence that the operation of lithotomy should be performed, if necessary: and it was only after the forms of binding the boy and bandaging his eyes were gone through, that he could be prevailed upon to confess his father had taught him to introduce these substances, which he had procured from coals, for the purpose of exciting commiseration for his pretended sufferings, and obtaining money from the charitably disposed; and, perhaps, in this instance, to have extorted money from the surgeon to conceal his ignorance, had he seriously attempted to perform any operation."

*Alvine Concretions.*—It sometimes occurs that bodies of a very anomalous kind are passed from the intestines; but the medical practitioner, by a careful examination of the substance, and a minute inquiry into the nature of all the ingesta, will frequently succeed in tracing their origin. Dr. Marcet, in his "Essay on Calculous Disorders," relates some interesting instances of this kind, which we shall notice in this place, in order to put the medical man on his guard when called upon to deliver his opinion upon such occasions. The first case is that of some concretions put into Dr. Marcet's hands by Sir Astley Cooper, and which had been discharged by a female patient, under circumstances which made it questionable whether they had proceeded from the rectum or from the urethra: they were, however, discovered to be pieces of undigested cheese formed into balls by the action of the intestines, or portions of caseous matter actually formed in the intestines from milk taken as nourishment by the person, and coagulated by the gastric juices into those undigestible masses. Another singular species of intestinal calculus was found by Dr. Marcet and Dr. Wollaston to be oat seeds, derived from the oaten cake which the patient had eaten. Dr. Marcet also describes a concretion which, by the assistance of Dr. Wollaston, he discovered to be those small woody knots which are often found in certain pears, and which the person had previously eaten. The last case which he relates is not less curious: a philosophical gentleman, of delicate health and disordered system, voided a number of small red globular bodies, each of which had in its centre two black opaque spots:

they were supposed to be peculiar animals connected with his disorder, but Dr. Wollaston soon satisfied himself that they were nothing but the spawn of lobsters, an extremely indigestible substance, of which the patient acknowledged to have eaten about the time he passed these bodies.—The author has deemed it necessary to introduce this subject under the present article; for, strange as it may appear, it not unfrequently happens, as Dr. Marcet has stated, that persons apparently respectable produce bodies, as having been voided, which are wholly supposititious.

*Abstinence from Food.*—Long fasting, or the power of refraining altogether from food for years, has been frequently the subject of imposition. The case of Ann Moore, of Tetbury, must be in the recollection of all our readers; and in the Philosophical Transactions two cases are recorded, in one of which a man is said to have taken nothing but water for eighteen years, with now and then, during a certain period of the year, a draught of clarified honey. But the case which has excited public interest in the greatest degree, is that of Elizabeth Canning, who, among other circumstances, pretended that she had been confined in a loft from Tuesday, the 2d of January, at four o'clock A.M. until Monday the 29th, at four P.M.; and that during this period she had had no sustenance, except about twenty-four pieces of bread, to the amount of a quartern loaf, a penny mince-pye, and between three and four quarts of water; and yet that, on the twenty-eighth day, she made her escape by jumping out of the window, and walked twelve miles in six hours without taking food. This story, incredible as it may appear, was actually believed by many persons, and popular clamour rose to a most indecent height: bills of indictment were preferred, and libels circulated, without example either as to number or virulence; and Mary Squires, an unfortunate old gipsey, was condemned to death for the robbery charged to have been committed previous to this alleged wanton imprisonment of the impostress Canning. One of the most interesting points in the evidence of these trials, (for there were several on different grounds,) was derived from the inspection of the linen of the impostress by an ingenious midwife, who observed that in twenty-eight days a menstrual period would probably have occurred, and yet there was no vestige of such an event to be traced on the linen:—thus may physiological circumstances often elucidate points apparently remote from medical cognizance.

*Deafness and Dumbness.*—Where the former of these maladies is alone simulated, the inspector will be able, with a little address, to detect the imposture: a sudden noise will frequently betray the patient, and an instance of this kind is related by Ambrose Paré. We may also contrive to communicate, in his presence, some circumstance in which he is greatly interested, and notice the effect of the intelligence upon his countenance, or upon his pulse. Where dumbness is only feigned, we should remember that the powers of articulation never leave a person without some cause, which medical inquiry must discover. It has been a question whether the absence of the tongue should be considered a sufficient reason for muteness: although we cannot dispute the validity of such a proof, it is necessary to know that cases are recorded where persons did very well without that organ; but we are inclined to believe, with Dr. Smith, that the muscles belonging to the tongue were, in such cases, not deficient. But these observations apply to instances of imposture, where deafness or dumbness have been singly simulated. Suppose a medical practitioner is called upon to examine a patient who declares himself to labour under the misfortune of congenital deafness, and consequent dumbness, what plan of investigation is he to pursue upon such an occasion? It must be admitted that, where this simulation is well performed, it becomes extremely difficult to detect it; but it requires so much art and perseverance, that few persons will be found capable of the deception. M. Sicard succeeded in the detection of a most accomplished impostor, by requiring him to answer a number of queries in writing; when the Abbé soon found that he spelt several words in compliance with their sound, instead of according to their established orthography,—by substituting, for instance, the *c* for the *q*; which at once enabled the Abbé to declare that it was impossible that he should have been deaf and dumb from his birth, because he wrote as we *hear*, and not (as in the case of the real deaf and dumb) as we *see*.

*Blindness.*—In cases of alleged amaurosis, the practitioner has generally relied upon the contractility of the pupil, as a test of vision; but Richter asserts that nothing positive can be drawn from the mobility or immobility of the iris, as sometimes the one and sometimes the other occurs. If, however, the pupil does not contract, we must think that the practitioner is authorised in concluding as to the existence of the disease. By unexpectedly reflecting the rays of the sun, by means of a mirror, upon the eye of the patient, we shall generally be able to discover any deception that may have been practised. Where short-sightedness is pleaded as a disqualification, the truth may be easily ascertained by inspection. The French adopted a very simple and ingenious mode of distinguishing the feigned myopes, who endeavoured to escape the conscription laws: they placed spectacles of various powers upon the persons to be examined, and suddenly bringing before their eyes a printed paper, the subject of which was wholly unknown to them, the facility with which the person read pointed out with tolerable accuracy the state of his vision. A myope, for instance, and none but a myope, could read fluently a paper brought close to his eyes, with concave glasses, and *vice versa*.

*Ophthalmia.*—This affection has been sometimes induced by the application of corrosive sublimate. If, says Dr. Hennen, in any suspected corps we find that the right eye is universally affected, it gives a reasonable ground to suppose that the deleterious substance has been put in preference into that eye, from design, or perhaps from the facility which the impostor derives from his right hand. A left-handed person will, for the same reason, inflict the injury on the left side.

*Ulcers, &c.*—External sores are constantly feigned by mendicants to obtain relief, or by soldiers to procure their discharge; and for this purpose various acrid applications, as well as pressure, have been resorted to. Galen detected an imposture of this kind, where a slave, in order to avoid accompanying his master on a long voyage, produced tumors in his knees by the application of *thapsus*. Ulcers, says Dr. Hennen, were formerly extremely prevalent in the army, and were often produced by various acrid substances; but, by the adoption of Mr. Baynton's practice, they are now rendered much more manageable. Where the ulcer is supposed to be excited by unfair means, surgeons are now in the habit of sealing the dressings, and so effectually preventing any improper tampering with them, without immediate discovery. Dr. Hennen says, "I had some time ago a case in a recruit, reported to be Pompholyx Diutinus, and resembling that species of bullæ in a very remarkable degree: after several weeks Dr. Bartlett, of the 88th regiment, into whose charge the man was at length transferred, detected a shining particle of the powder of cantharides adhering to an unctuous dressing, which had been purposely applied loosely to the limb, in order that the patient might not be prevented from managing his case in his own way." On some occasions the *ranunculus flammula* has been employed for these iniquitous purposes; in others, verdigris, or a copper coin, has been bound tight on the sore.

*Hernia* has been sometimes simulated by blowing air into the cellular membrane; and *Prolapsus Ani* has been successfully imitated by introducing a foreign gut into the rectum.

### *Of the Adulteration of Food.*

Although it is generally acknowledged that the representations of the ephemeral writer who lately excited so much public notice, were no less preposterous than the symbols which decorated his volume, yet it cannot be denied that a great part of our daily food, and a still greater portion of our luxuries, are the constant objects of fraudulent adulteration: and what reasonable hope can be entertained of any amendment while the temptations remain so excessive, the detection so difficult, and the punishment so inadequate to the crime; or, above all, while the trouble and expense of prosecution continue to be so disproportioned to the injury sustained by an individual, as to prevent his seeking redress through the ordinary channels of the law? These observations, perhaps, apply with greater force to the adulteration of articles not subject to the revenue duties of excise or customs, such as bread, milk, &c. Against the substitution or sophistication of those whose sale enriches the treasury, we have numerous enactments; and, were we to form our judgment from them alone, we should conclude that fraudulent adulterations were rather deprecated as offences against the revenue, than against the health of the citizen. It is, however, important to remark, that, if the health of any person be impaired in consequence of the act of another, as by selling him bad wine, which injures the party's health, an action (viz. a trespass on the case,) will lie.

The adulteration of bread is specially prohibited by several statutes: the 31 Geo. II. c. 29, entitled 'An Act for the due making of Bread, and to regulate the price and assize thereof, and to punish persons who shall adulterate meal, flour, or bread," after reciting the 51 Hen. III. and 8 Anne, c. 18, and making various regulations as to the assize, enacts that bread made for sale shall be of meal or flour, and that no alum, or preparation or mixture in which alum shall be an ingredient, or any other mixture or ingredient whatsoever, (except only the genuine meal or flour which ought to be put therein, and common salt, pure water, eggs, milk, yeast, and barm, or such leaven as shall at any time be allowed to be put therein by the court or magistrates.) And that no person shall knowingly put into any corn, meal, or flour, which shall be ground, dressed, bolted, or manufactured for sale, any ingredient, mixture, or thing whatsoever, or shall knowingly sell any thing which shall not be real and genuine meal or flour of the grain the same shall import to be.

With respect to the manufacture of malt liquors, especially porter, it is wholly under the jurisdiction of the excise, and yet there is no article of diet which has so universally the credit of being adulterated, and that too with drugs of the most noxious quality. We have now lying before us "Minutes taken (in session 1818) before the Committee of the House of Commons, to whom the petition of several inhabitants of London and its vicinity, complaining of the high price and inferior quality of Beer, was referred, to examine the matter thereof, and report the same, with their observations thereupon, to the House. Ordered, by the House of Commons, to be printed, 8 April, 1819." From this it very clearly appears, that the illegal addition of various drugs is commonly practised in the breweries: but we are nevertheless inclined to believe that the more extensive and serious frauds of this description are not carried on in the cauldrons of the brewer, but in the barrels of the publican.

The adulteration of milk has furnished another object of popular clamour, but we are inclined to believe that its dilution with water is the only fraud ever committed with respect to it. Chalk, if added, would be so easily detected, and would answer the purpose so clumsily, that we may very safely consider such a charge against the London milk-venders as entirely groundless.

In order to assay the quality of milk, several different instruments have been proposed. Mr. Dicas, mathematical instrument maker in Liverpool, invented for this purpose an instrument which he termed a *lactometer*, and which ascertains the richness of milk from its specific gravity compared with water. Mr. Edmund Davy, of Cork, has lately made a very interesting application of the hydrometer, to ascertain the quality of skimmed milk.

